# Application of a Modified First-Order Plate Theory to Structural Analysis of Sensitive Elements in a Pyroelectric Detector

**DOI:** 10.3390/mi15081012

**Published:** 2024-08-06

**Authors:** Mengmeng Lian, Cuiying Fan, Xiaohan Zhan, Minghao Zhao, Guoshuai Qin, Chunsheng Lu

**Affiliations:** 1 School of Mechanics and Safety Engineering, Zhengzhou University, Zhengzhou 450001, China; 15294688900@163.com (M.L.); zzuzxiaohan@163.com (X.Z.); memhzhao@zzu.edu.cn (M.Z.); 2 School of Electromechanical Engineering, Henan University of Technology, Zhengzhou 450001, China; gsqin@haut.edu.cn; 3 School of Civil and Mechanical Engineering, Curtin University, Perth, WA 6845, Australia

**Keywords:** pyroelectric detector, pyroelectricity, piezoelectricity, first-order plate theory, electric potential, sensitive element

## Abstract

Pyroelectric materials, with piezoelectricity and pyroelectricity, have been widely used in infrared thermal detectors. In this paper, a modified first-order plate theory is extended to analyze a pyroelectric sensitive element structure. The displacement, temperature, and electric potential expand along the thickness direction. The governing equation of the pyroelectric plate is built up. The potential distributions with upper and lower electrodes are obtained under different supported boundary conditions. The corresponding numerical results of electric potential are consistent with those obtained by the three-dimensional finite element method. Meanwhile, the theoretical results of electric potential are close to that of experiments. The influence of supported boundary conditions, piezoelectric effect, and plate thickness are analyzed. Numerical results show that the piezoelectric effect reduces the electric potential. The thickness of the pyroelectric plate enhances the electric potential but reduces the response speed of the detector. It is anticipated that the pyroelectric plate theory can provide a theoretical approach for the structural design of pyroelectric sensitive elements.

## 1. Introduction

Because pyroelectric piezoelectric materials have pyroelectricity and piezoelectricity simultaneously, infrared thermal detectors can be prepared to realize the conversion of light, heat, and electricity [1]. The pyroelectric detector is pivotal for gas detection, human body temperature measurement, fire alarms, and other applications [2,3]. Several factors affect the detector performance [4], including ambient temperature, working load, and the manufacturing process [5]. Therefore, it is of practical significance to investigate the performance of pyroelectric materials under various temperatures. Lithium tantalite (LiTaO_3_), as an important pyroelectric material [6], is the most suitable material for sensitive elements in surface acoustic wave devices, thermoelectric detectors, and electro-optical modulation [7,8]. However, LiTaO_3_ has the piezoelectric property [9], which would influence its thermo-electric effect. Thus, it is necessary to explore the mechanical-thermal-electric coupling properties of sensitive elements.

The sensitive element in a pyroelectric detector is a laminar structure, with upper and lower surfaces covered by uniform electrodes [10]. Generally, the thin plate theory can be utilized to analyze the mechanical, thermal, and electrical behaviors of a sensitive element structure. In elastic plate theories, different hypotheses are introduced on the displacement field along the thickness direction, describing deformation of a plate [11]. For example, a plane-parallel capacitor was used to establish the model of a piezoelectric plate with two complete surface electrodes [12]. It implies that the electric field inside a piezoelectric layer is constant and independent of plane coordinates, i.e., there is no electric and mechanical coupling. This theory has also been utilized in analyzing the vibration characteristics of a piezoelectric semiconductor cantilever [13], piezoelectric semiconductor plates [14], and the pre-buckling behavior of piezoelectric semiconductor plates [15]. Taking the piezoelectric effect into account, the same assumptions were applied to describe the distribution of electric potential along the thickness direction, such as the third-order power series expansion [16,17], sinusoidal functions [18,19], cosine functions [20], and the combination of polynomial and cosine functions [21]. Further, a modified first-order plate theory was proposed, in which a quadratic distribution was utilized to simulate the distribution of electric potential in a piezoelectric plate [22]. In contrast to other assumptions, the expansion terms of an electric potential, an electric field, and its differential have definite and clear physical meanings. 

There are two main processes from excitation to response in a pyroelectric detector. Firstly, the sensitive element absorbs radiation and leads to the temperature increasing. Then, due to the pyroelectric effect, the temperature change generates pyroelectric voltage [23]. Recently, technologies have been well developed for manufacturing absorption layers in pyroelectric infrared detectors with a high radiation absorption rate. For instance, Zhao et al. [24] fabricated a sensitive carbon black absorption layer with an impressive absorption rate of 0.94. Upon exposure to radiation, the sensitive element absorbs the incoming radiation and subsequently acquires the heat flux. Therefore, considering pyroelectric materials as a sensitive element, the temperature change induces the significant electric field induced by the pyroelectric effect.

The classical plate theory does not consider the transverse shear deformation, which could induce a certain amount of error. Thus, we chose the modified first-order piezoelectric plate theory to study the pyroelectric plate. Then, the theory is utilized for structural analysis of a sensitive element in a pyroelectric detector, where displacement is expanded in the first order, and the electric potential and temperature in the second order along the thickness direction. Finally, a theoretically optimal structure is suggested, which can be instructive for the design of pyroelectric infrared detectors.

## 2. Basic Equations

Let us consider a homogeneous pyroelectricity plate. The equilibrium equations [25] under steady state conditions are

(1) $$\sigma_{ij,i}=0,\;D_{i,i}=0,\;h_{i,i}=0,\;\left(i,j=1,2,3\right)$$

where *σ_ij_* is the stress, *D_i_* is the electric displacement vector, and *h_i_* is the heat flux. The subscript, i.e., a comma followed by a letter, donates the partial differentiation with respect to the coordinate associated with the letter. In consideration of pyroelectric effects, the piezoelectric constitutive equations are

(2) $$\sigma_{ij}=c_{ijkl}\varepsilon_{kl}-e_{ijk}E_{k},\;\;D_{i}=e_{ijk}\varepsilon_{jk}+k_{ij}E_{j}+p_{i}\theta ,\;\;h_{i}=-\beta_{ij}\theta_{,j},$$

where *c_ijkl_*, *e_ijk_*, *κ_ik_*, *p_i_* and *β_ij_* are the elastic, piezoelectric, dielectric, pyroelectric, and heat conductivity coefficients, respectively. Here the strain tensor *ε_ij_*, the electric filed *E_i_*, and the temperature gradient $\Theta_{j}$ are defined as

(3) $$\varepsilon_{ij}=\frac{1}{2}\left(u_{i,j}+u_{j,i}\right),\;\;E_{i}=-\varphi_{,i},\;\;\Theta_{j}=\theta_{,j},$$

## 3. The Modified First-Order Plate Theory

**Figure 1:**
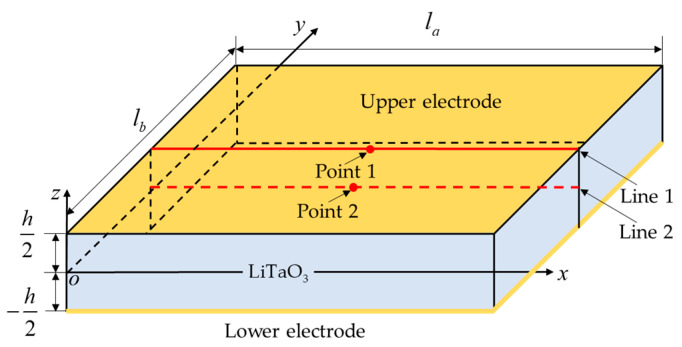
Illustration of a plane coordinate system for the piezoelectric plate, in which two typical lines and points are respectively chosen as follows: Line 1: *x* ∈ [0, *l_a_*], *y* = *l_b_*/2, *z* = *h*/2; Line 2: *x* ∈ [0, *l_a_*], *y* = *l_b_*/2, *z* = 0; Point 1: (*x*,*y*,*z*) = (*l_a_*/2, *l_b_*/2, *h*/2); Point 2: (*x*,*y*,*z*) = (*l_a_*/2, *l_b_*/2, 0).

As illustrated in Figure 1, the coordinate plane *oxy* is located on the central plane of a pyroelectricity plate. The polarization direction is along the plate thickness (*z*) direction. The structure is composed of an intermediate layer of LiTaO_3_, and upper and lower electrodes. The thicknesses of the upper and lower electrodes are much smaller than that of the piezoelectric plate. Therefore, the effect of the electrodes can be omitted.

When the length *l_a_* and width *l_b_* of a plate are much larger than the thickness *h*, the stress relaxation approximation, *σ*_33_ = 0, can be introduced [26]. Then, it is obtained as

(4) $$\varepsilon_{33}=-\frac{1}{c_{3333}}\left[c_{33kl}\varepsilon_{kl}-e_{33k}E_{k}-c_{3333}\varepsilon_{33}\right].$$

Substituting Equation (4) into Equation (2), we have

(5) $$\sigma_{ij}={\bar{c}}_{ijkl}\varepsilon_{kl}-{\bar{e}}_{ijk}E_{k},\;\;D_{i}={\bar{e}}_{ikl}\varepsilon_{kl}+{\bar{\kappa }}_{ik}E_{k}+{\bar{p}}_{i}\theta ,$$

where the equivalent material constants are defined as

(6) $$\begin{array}{l} {\bar{c}}_{ijkl}=c_{ijkl}-c_{ij33}c_{33kl}/c_{3333},\;\;{\bar{e}}_{kij}=e_{kij}-e_{k33}c_{33ij}/c_{3333},\\ {\bar{\kappa }}_{ik}=\kappa_{ik}+e_{i33}e_{33k}/c_{3333},\;\;\;{\bar{p}}_{i}=p_{i}+e_{i33}\lambda_{3333}/c_{3333}. \end{array}$$

Due to the influence of transverse shear deformation and piezoelectric effect, the accuracy requirements cannot be satisfied by the first-order expansion of an electric field. Thus, Lian et al. [22] introduced a modified first-order plate theory for a laminated piezoelectric plate. Here, a similar method is adopted, with the first-order expansion for the displacement and the second-order expansion for the electric potential and temperature, that is

(7) $$\begin{array}{l} u=u^{\left(0\right)}\left(x,y\right)+zu^{\left(1\right)}\left(x,y\right),\\ v=v^{\left(0\right)}\left(x,y\right)+zv^{\left(1\right)}\left(x,y\right),\\ w=w^{\left(0\right)}\left(x,y\right),\\ \varphi \left(x,y,z\right)=\varphi^{\left(0\right)}\left(x,y\right)+z\varphi^{\left(1\right)}\left(x,y\right)+z^{2}\varphi^{\left(2\right)}\left(x,y\right),\\ \theta \left(x,y,z\right)=\theta^{\left(0\right)}\left(x,y\right)+z\theta^{\left(1\right)}\left(x,y\right)+z^{2}\theta^{\left(2\right)}\left(x,y\right), \end{array}$$

where *u*^(0)^ and *v*^(0)^ are the displacements of points in the plane of a plate, and *u*^(1)^ and *v*^(1)^ are the rotation of points in the middle plane. *φ*^(0)^ is the electric potential at a point on the plate’s reference plane, while *φ*^(1)^ and *φ*^(2)^ are the first- and second-order expansion terms of the potential, respectively. Here it is worth noting that the temperature at any point in the plate can be characterized by three parameters: the temperature itself, its gradient, and the higher-order expansion terms, denoted as *θ*^(0)^, *θ*^(1)^, and *θ*^(2)^, respectively.

Based on Equation (7), the strain field, electric field, and temperature gradient field can be obtained as

(8) $$\begin{array}{l} \varepsilon_{ij}^{\left(a\right)}=\frac{1}{2}\left[u_{i,j}^{\left(a\right)}+u_{j,i}^{\left(a\right)}+\left(a+1\right)\left(\delta_{3j}u_{i}^{\left(a+1\right)}+\delta_{3i}u_{j}^{\left(a+1\right)}\right)\right],\\ E_{i}^{\left(a\right)}=-\left[\varphi_{i}^{\left(a\right)}+\left(a+1\right)\delta_{3i}\varphi^{\left(a+1\right)}\right],\\ {\bar{\Theta }}_{j}^{\left(a\right)}=\theta_{,j}^{\left(a\right)}+\left(a+1\right)\delta_{3j}\theta^{\left(a+1\right)}, \end{array}\mathrm{note}:\{\begin{array}{c} \delta_{ij}=1,\;i=j\\ \delta_{ij}=0,\;i\neq j \end{array}$$

Integrating Equation (1) across the plate thickness and establishing the internal force equivalence, the equilibrium equations of the Mindlin plate are derived, that is

(9a) $$\sigma_{ij,i}^{\left(m\right)}-m\sigma_{3j}^{\left(m-1\right)}+t_{j}^{\left(m\right)}=0,\;\;m=0,1$$

(9b) $$D_{i,i}^{\left(0\right)}+d^{\left(0\right)}=0,\;\;\;h_{i,i}^{\left(0\right)}+{\tilde{H}}^{\left(0\right)}=0,$$

where

(10) $$\begin{array}{l} \sigma_{ij}^{\left(m\right)}=\sum_{a=0}^{\infty }\left(\left(\int_{-h/2}^{h/2}z^{m}z^{a}{\bar{c}}_{ijkl}dz\right)\varepsilon_{kl}^{\left(a\right)}-\int_{-h/2}^{h/2}z^{m}z^{a}{\bar{e}}_{ijk}dzE_{k}^{\left(a\right)}\right),\\ D_{i}^{\left(0\right)}=\sum_{a=0}^{1}\left(\int_{-h/2}^{h/2}z^{a}{\bar{e}}_{ijk}dz\right)\varepsilon_{kl}^{\left(a\right)}+\sum_{b=0}^{2}\left[\left(\int_{-h/2}^{-h/2}z^{b}{\bar{\kappa }}_{ik}dz\right)E_{k}^{\left(b\right)}+\left(\int_{-h/2}^{h/2}z^{b}p_{i}dz\right)\theta^{\left(b\right)}\right],\;i=1,2\\ h_{i}^{\left(0\right)}=\sum_{a=0}^{1}\left(\int_{-h/2}^{h/2}\beta_{ij}z^{a}dz\right)\theta_{,j}^{\left(a\right)},\;\;\;\\ t_{j}^{\left(m\right)}={\left[\sigma_{3j}z^{m}\right]}_{-h/2}^{h/2},\;\;d^{\left(0\right)}={\left[D_{3}\right]}_{-h/2}^{h/2},\;\;{\tilde{H}}^{\left(0\right)}={\left[h_{3}\right]}_{-h/2}^{h/2},\;\;\;\;\;\;\;\;\; \end{array}$$

### 3.1. Elastic Boundary Conditions

Based on the support method of a detector sensitive element, there are the following four displacement constraints.

Four-side clamped:

(11a) $$x=0\;\mathrm{and}\;l_{a},\;\ldots \;u^{\left(0\right)}=0,v^{\left(0\right)}=0,w^{\left(0\right)}=0,u^{\left(1\right)}=0,v^{\left(1\right)}=0,$$

(11b) $$y=0\;\mathrm{and}\;l_{b},\;\ldots \;u^{\left(0\right)}=0,v^{\left(0\right)}=0,w^{\left(0\right)}=0,u^{\left(1\right)}=0,v^{\left(1\right)}=0.$$

Four-side simply supported:

(12a) $$x=0\;\mathrm{and}\;l_{a},\;\ldots \;v^{\left(0\right)}=0,\;w^{\left(0\right)}=0,\;v^{\left(1\right)}=0,$$

(12b) $$y=0\;\mathrm{and}\;l_{b},\;\ldots \;u^{\left(0\right)}=0,\;w^{\left(0\right)}=0,\;u^{\left(1\right)}=0.$$

Two-side clamped:

(13) $$x=0\;\mathrm{and}\;l_{a},\;\ldots \;u^{\left(0\right)}=0,v^{\left(0\right)}=0,w^{\left(0\right)}=0,u^{\left(1\right)}=0,v^{\left(1\right)}=0.$$

Four-point simply supported:

(14a) $$x=0,y=0\;\mathrm{and}\;l_{b},\;\ldots \;u^{\left(0\right)}=0,v^{\left(0\right)}=0,w^{\left(0\right)}=0,$$

(14b) $$x=l_{a},y=0\;\mathrm{and}\;l_{b},\;\ldots \;u^{\left(0\right)}=0,v^{\left(0\right)}=0,w^{\left(0\right)}=0,$$

### 3.2. Thermal Boundary Conditions

When a sensitive element absorbs radiation, the temperature in the pyroelectric detector increases, resulting in a temperature differential on the upper and lower surfaces. Consequently, the thermal boundary conditions of a detector [27] are

(15a) $$\theta =\theta_{0}+\Delta \theta ,\;\ldots \ldots \;\mathrm{at}\;z=\frac{h}{2}$$

(15b) $$\theta =\theta_{0},\;\ldots \ldots \;\mathrm{at}\;z=-\frac{h}{2}$$

where Δ*θ* is the temperature difference between upper and lower surfaces of the plate, and *θ*_0_ is the reference temperature, *θ*_0_ = 300 K.

### 3.3. Electrical Boundary Conditions

The electric potentials are uniformly distributed in a pyroelectricity plate with upper and lower electrodes. If the lower electrode is grounded, the electric boundary conditions are

(16a) $$\varphi =V_{1},\;\ldots \ldots \;\mathrm{at}\;z=\frac{h}{2}$$

(16b) $$\varphi =0,\;\ldots \ldots \;\mathrm{at}\;z=-\frac{h}{2}$$

(16c) $$D_{i}^{\left(0\right)}n_{i}=0.\;\ldots \;\mathrm{at}\;x=0,l_{a}\;\mathrm{or}\;y=0,l_{b}$$

However, the upper surface potential V_1_ of the plate is an unknown constant for a pyroelectricity detector. That is, one supplementary equation is needed to maintain electrical neutrality. According to Gauss’s theorem, the upper and lower surface electrical displacements satisfy

(17) $$\int_{S_{up}}D_{3}dS-\int_{S_{down}}D_{3}dS=\int_{S}\left[{\bar{e}}_{31}\left(hu_{,x}^{\left(1\right)}+hv_{,y}^{\left(1\right)}\right)-\frac{8{\bar{k}}_{33}}{h}\left(\frac{V_{1}}{2}-\varphi^{\left(0\right)}\right)+{\bar{p}}_{3}h\theta^{\left(1\right)}\right]dS=0,$$

where *S_up_* and *S_down_* are the areas of the upper and lower surfaces of the plate, with *S* = *S_up_* = *S_down_*. Obviously, such a mixed boundary condition is nonlinear. The thermal boundary equations on the upper and lower surfaces in Equation (15), along with the electric boundary equations in Equations (16b) and (17), the stress equations in Equation (9a), and the electric displacement equations and heat flow equations in Equation (9b), are solved with MATLAB R2018a and the Partial Differential Equations module of COMSOL 5.3a.

It is worth noting that, in the classical plate theory, the expansion terms in Equation (7) can be expressed by

(18) $$u^{\left(1\right)}=-\frac{\partial w}{\partial x},\;v^{\left(1\right)}=-\frac{\partial w}{\partial y},\;\varphi^{\left(1\right)}=\frac{\partial \varphi }{\partial z},\;\theta^{\left(1\right)}=\frac{\partial \theta }{\partial z},\;\varphi^{\left(2\right)}=0,\;\theta^{\left(2\right)}=0$$

This can lead to automatic satisfaction of the proposed conditions of electric neutrality (see Equation (17)). Thus, it is consistent with the hypothesis of parallel plate capacitors. Meanwhile, Equation (18) shows that the distribution of electric potential along thickness is no longer in the form of a quadratic function. That is, such a hypothesis does not accurately reflect the actual potential distribution along thickness in pyroelectric detectors. Therefore, the first-order plate theory was adopted in this paper.

## 4. Verification of Plate Theory and Discussion

### 4.1. Verification of 3D Finite Element

**Figure 2:**
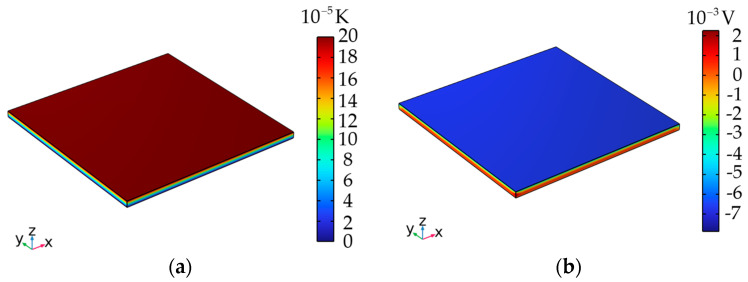
The distributions of (**a**) the temperature Δ*θ* and (**b**) the electric potential *φ* in a four-side clamped plate.

LiTaO_3_ was selected as the pyroelectric thin plate with the length and width of *l_a_* = *l_b_* = 3 × 10^−3^ m. The corresponding material constants are listed in Table 1. Due to the pyroelectric effect, an external temperature is applied to the plate, generating an electric potential difference between its upper and lower surfaces. Figure 2 depicts typical three-dimensional (3D) cloud images of the temperature *θ* and electric potential *φ* in the plate under the four-side clamped condition, with a surface temperature difference of 2 × 10^−4^ K. In a pyroelectric plate subjected to a thermal load, the heat propagates along the thickness of the plate, resulting in the formation of an isothermal surface that is distributed perpendicularly to the *z*-axis, as shown in Figure 2a. The corresponding electric potential exhibits a similar distribution to the temperature profile within the plate, as shown in Figure 2b. 

**Figure 3:**
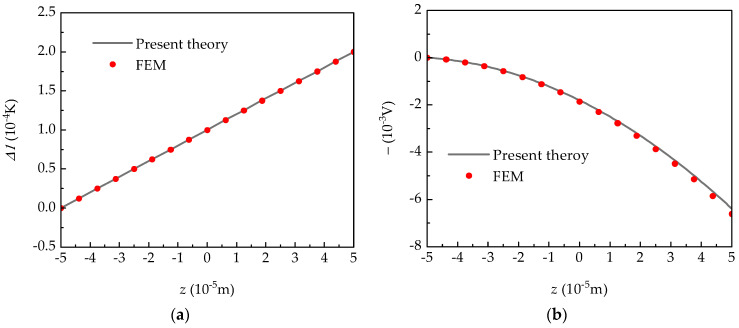
The distributions of (**a**) temperature and (**b**) electric potential along the plate thickness under a four-side clamped plate.

Under the same temperature, Figure 3 is a more comprehensive illustration of the distributions of temperature and electric potential across plate thickness under the four-side clamped condition, where the computing module of floating potential in COMSOL 5.3a Multiphysics was used in the 3D finite element method (FEM). It is shown that the theoretical results of the pyroelectric plate have a high accuracy. Meanwhile, it is noteworthy that temperature exhibits an approximate linear distribution along the plate thickness, and electric potential demonstrates a strong nonlinear variation. Such a nonlinear phenomenon of electric potential also appears under the other three supported conditions. This confirms the accuracy of Equation (7) for the temperature and electric potential hypothesis and the rationality of electric and thermal boundary conditions.

**Figure 4:**
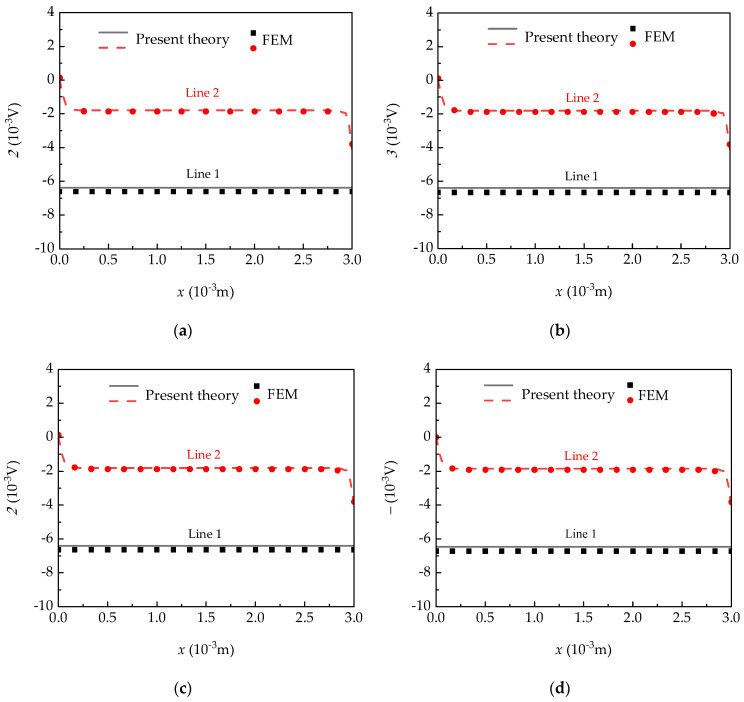
The distributions of electric potential along the *x*-axis when Δ*θ* = 2 × 10^−4^ K for (**a**) four-side clamped; (**b**) four-side simply supported boundary conditions; (**c**) two-side clamped; and (**d**) four-point simply supported boundary conditions.

The electric potential obtained by the plate theory was compared to those from the 3D FEM under four elastic-supported boundary conditions, as shown in Figure 4. The electric potential along Line 1 is constant, and along Line 2, it is also constant except at the plate edge of a central plane. Table 2 lists the specific numerical values and numerical errors of the electric potential at Points 1 and 2 under different supported conditions. These results agree with that derived from the solutions using the 3D finite element approach. Therefore, the correctness is reconfirmed on the theoretical derivation of the pyroelectric plate theory. Meanwhile, it is seen that the small influence of differently supported conditions can be ignored.

**Figure 5:**
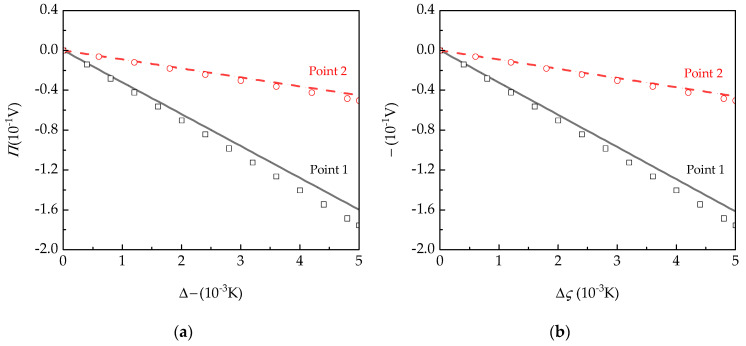
The influence of Δ*θ* on electric potential at Points 1 and 2 obtained from the present theory (line), and Ref. [28] (hollow dot) under (**a**) four-side clamped and (**b**) four-point simply supported boundary conditions.

Figure 5 analyzed the influence of pyroelectric behaviors on the electric potential with increasing temperature. It is observed that the overall potential increases linearly. Specifically, the growth rate of potential at Point 1 on the upper surface surpasses that of Point 2 on the middle plane. This implies that the response time of the pyroelectric plate detector becomes quicker under a higher external temperature, leading to a stronger generated signal. Meanwhile, the influence of support states on electric potential can also be ignored.

Without considering the piezoelectric property [28], the corresponding electric potential is shown in Figure 5. It is obvious that, when considering the piezoelectric effect, the response electric potential of the pyroelectric plate decreases under identical conditions. As the temperature load rises, the inhibitory impact of the piezoelectric effect on the plate becomes increasingly pronounced. Based on the present theory, under Δ*θ* = 5 × 10^−3^ K, the electric potential is reduced by 10% under the four-side clamped boundary condition due to the piezoelectric effect. Therefore, it is necessary to consider the influence of piezoelectric effects under the high temperature difference.

### 4.2. Verification of Experimental Results

**Figure 6:**
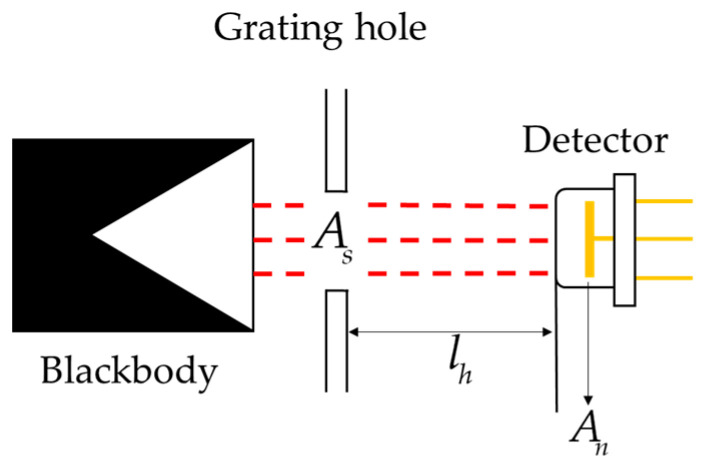
Illustration of the process of blackbody radiation in a pyroelectric detector.

Given that the sensitive element absorbs the incoming radiation and generates heat, the heat flux *Q_t_* can be represented as

(19) $$Q_{t}=\gamma RA_{n},$$

where *γ* is the absorption rate of incident radiation in the absorption layer, *A_n_* is the detector area, and *R* is the blackbody irradiance. In the field of infrared detector production, the blackbody furnace with a furnace temperature of 500 K is usually used as the standard radiation source. As illustrated in Figure 6, the blackbody irradiance can be calculated by [29]

(20) $$R=\alpha \frac{\delta S_{t}\left({\theta_{H}}^{4}-\theta_{0}^{4}\right)A_{s}}{\pi {l_{h}}^{2}},$$

where α is a modulation factor that is controlled by a chopper. It represents the proportion of blackbody radiation through the chopper. Typically, radiation is blocked half of the time, so α = 0.5 is usually used in calculations. *δ* is the effective emissivity of blackbody radiation source, *S_t_* is the Stefan–Boltzmann constant, *θ_H_* and *θ_0_* are the blackbody temperature and ambient temperature, respectively, *A_s_* is the grating area of the blackbody radiation source, and *l_h_* is the distance between light from the blackbody source and the detector. 

The temperature rise rate affects its sensitivity. There is a positive correlation between the response speed of the detector and the temperature rise rate of the sensitive element. In terms of the heat conduction theory [30,31], we have

(21) $$d\theta_{m}=\frac{Q_{t}}{cm}dt=\frac{Q_{t}}{c\rho A_{n}h}dt,$$

where *c* is the constant pressure heat capacity of a pyroelectric material, *m* is the weight of the pyroelectric material, *ρ* is the material density, *h* is the thickness of the sensitive element, *dθ_m_* is the temperature rise of the upper surface of the structure, and *t* is the time. Therefore, the temperature and the heat flux are in linear relation. 

**Figure 7:**
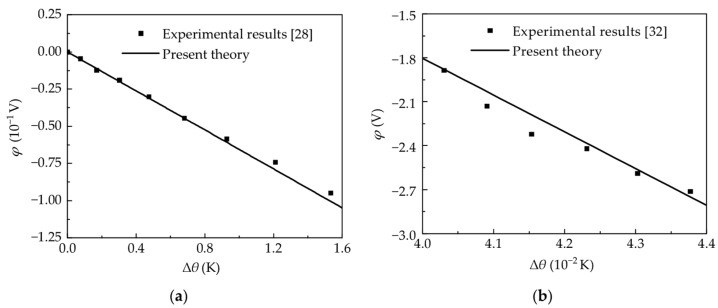
Comparison between theorical and experimental potentials versus temperature on the upper surface in (**a**) PZT/PU composite material and (**b**) lithium tantalite.

Let us consider a lead zirconate titanate/castor oil-based polyurethane (PZT/PU) composite material plate with dimensions of 3 × 3 × 0.08 mm^3^ [28]. A light beam emitted from an unmodulated halogen lamp was focused on the sample fixed in the sensor chamber. The voltage from the temperature sensor was measured by a lock-in amplifier. Figure 7a illustrates the curve depicting the electric potential in relation to the change of temperature. In Ref. [32], a laser was used as the signal source and it was converted into a modulated square wave with the modulation factor *α* = 0.5 through a modulation technology. These experimental results for lithium tantalate tablets are compared with simulation results, as shown in Figure 7b, where lithium tantalite tablets with the same dimensions of 3 × 2 × 0.2 mm^3^ are used. Here it is worth noting that, although there seems to be a linear trend in simulations, electric potential increases nonlinearly with temperature. The theoretical results of electric potential are close to that of experiments. Figure 7a,b also prove the correctness and accuracy of theoretical results.

## 5. Optimization of Plate Thickness

**Figure 8:**
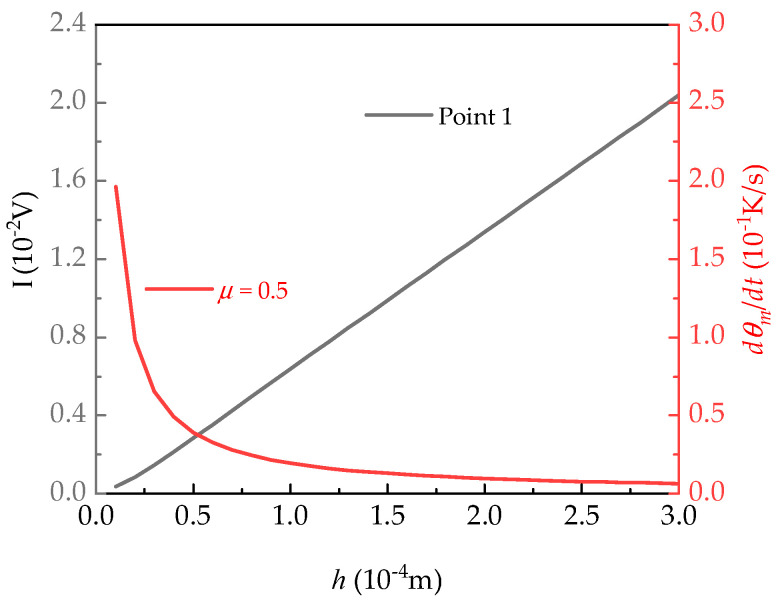
Influence of the plate thickness *h* on electrical potential and change rate of temperature.

To optimize the thickness of lithium tantalite tablets as sensitive elements in a pyroelectric detector, the influence of the radiation process should be considered. The corresponding constants during radiation are listed in Table 3. As shown in Figure 8, it is evident that, as plate thickness increases, pyroelectric electric potential increases linearly. However, taking the sensitivity of a sensor into account, we also investigated the influence of thickness on the sensitivity (see Figure 8). As the thickness of a pyroelectric plate increases, the rate of temperature change *dθ_m_*/*dt* decreases [33]. In particular, when the plate thickness is less than 100 μm, the response rate increases dramatically. Therefore, an excessively thick or thin plate is not conducive to enhancing the performance of a detector, and the appropriate plate thickness is about 50 μm.

## 6. Conclusions

In this paper, a modified first-order pyroelectric plate theory has been presented to analyze sensitive components. The semi-analytical solutions of electric potential distributions obtained under different supported boundary conditions are consistent with 3D finite element simulations and available experimental results. The main conclusions can be drawn as follows:

(1) The numerical results indicate that there is little influence of elastic boundary conditions on electric potential.
(2) As temperature generated by the surface of a sensitive element increases, the influence of the piezoelectric property strengthens. However, the piezoelectric property can reduce the response electric potential of a pyroelectric plate.
(3) A thicker plate results in a higher pyroelectric voltage, but it decreases the response speed of the detector response.

It is expected that the present theory will offer a valuable tool for analyzing the sensitive element performance in pyroelectric detectors and provide a theoretical foundation for optimizing the detector efficiency.

## Figures and Tables

**Table 1:** The material parameters of lithium tantalite [6].

Elastic Stiffness (10^10^ N m^−2^)	Piezoelectric Constant (C m^−2^)	Dielectric Constant (10^−10^ F m^−1^)	Pyroelectric Coefficient (10^−4^ C m^−2^ K^−1^)	Thermal Conductivity (W m^−1^ K^−1^)
*c*_1111_ = 23.30	*e*_113_ = 0.08	*κ*_11_ = 3.61	*p*_1_ = −2.3	*β*_1_ = 46
*c*_1122_ = 4.69	*e*_333_ = 1.88	*κ*_33_ = 3.83	*p*_3_ = −2.3	*β*_3_ = 46
*c*_1133_ = 8.02	*e*_311_ = 2.30			
*c*_3333_ = 27.54				

**Table 2:** Numerical values of potential under different support states.

Supported State	Present Theory		FEM		Upper Surface Error (%)	Median Plane Error (%)
	Point 1 Potential (10^−3^ V)	Point 2 Potential (10^−3^ V)	Point 1 Potential (10^−3^ V)	Point 2 Potential (10^−3^ V)		
Four-side clamped	−6.39	−1.80	−6.61	−1.85	−3.32	−2.70
Four-side simply supported	−6.39	−1.81	−6.66	−1.88	−4.05	−3.72
Two-side clamped	−6.39	−1.80	−6.64	−1.87	−3.77	−3.74
Four-point simply supported	−6.46	−1.84	−6.71	−1.91	−3.72	−3.66

**Table 3:** The constant parameters in the radiation process.

Parameter	Symbol	Value	Unit
Modulation factor	*α*	0.5	–
Effective emissivity	*δ*	0.99	–
The distance between light and detector	*l_h_*	0.1	m
Grating area	*A_s_*	7.9 × 10^−5^	m^2^
Heat capacity at constant pressure	*c*	250	J kg^−1^ K^−1^
Density	*ρ*	7450	kg m^−3^
Stefan–Boltzmann constant	*S_t_*	5.67 × 10^−8^	W m^−2^ K^−4^
Blackbody temperature	*θ_H_*	500	K
Ambient temperature	*θ*_0_	293.15	K

## Data Availability

The data that support the findings of this study are available from the corresponding author upon reasonable request.
